# MiR-10a, miR-15a, let-7a, and let-7g expression as stress-relevant biomarkers to assess acute or chronic psychological stress and mental health in human capillary blood

**DOI:** 10.1007/s11033-023-08467-5

**Published:** 2023-05-17

**Authors:** Ulrike D. B. Krammer, Mariam L. Lerch, Alexander G. Haslberger, Berit Hippe

**Affiliations:** 1 Department of Nutritional Science, University of Vienna, 1090 Vienna, Austria; 2 HealthBioCare GmbH, 1090 Vienna, Austria

**Keywords:** Psychological distress, Mental health, Biomarkers, Epigenetics, miRNAs

## Abstract

**Background:**

Psychological stress, as an important cofactor in the development of many acute and chronic diseases, is crucial for general health or well-being, and improved markers are needed to distinguish situations of progressive pathological development, such as depression, anxiety, or burnout, to be recognized at an early stage. Epigenetic biomarkers play an important role in the early detection and treatment of complex diseases such as cancer, and metabolic or mental disorders. Therefore, this study aimed to identify so-called miRNAs, which would be suitable as stress-related biomarkers.

**Methods and Results:**

In this study, 173 participants (36.4% males, and 63.6% females) were interviewed about stress, stress-related diseases, lifestyle, and diet to assess their acute and chronic psychological stress status. Using qPCR analysis, 13 different miRNAs (miR-10a-5p, miR-15a-5p, miR-16-5p, miR-19b-3p, miR-26b-5p, miR-29c-3p, miR-106b-5p, miR-126-3p, miR-142-3p, let-7a-5p, let-7g-5p, miR-21-5p, and miR-877-5p) were analyzed in dried capillary blood samples. Four miRNAs were identified, miR-10a-5p, miR-15a-5p, let-7a-5p, and let-7g-5p (*p* < 0.05), which could be used as possible candidates for measuring pathological forms of acute or chronic stress. Let-7a-5p, let-7g-5p, and miR-15a-5p (*p* < 0.05) were also significantly higher in subjects with at least one stress-related disease. Further, correlations were identified between let-7a-5p and meat consumption (*p* < 0.05) and between miR-15a-5p and coffee consumption (*p* < 0.05).

**Conclusion:**

The examination of these four miRNAs as biomarkers using a minimally invasive method offers the possibility of detecting health problems at an early stage and counteracting them to maintain general and mental health.

**Supplementary Information:**

The online version contains supplementary material available at 10.1007/s11033-023-08467-5.

## Introduction

Acute and chronic psychological stress leads to complex changes in physiological systems that, besides behavior, also affects inflammatory, cellular, and metabolic processes in the human body. It has been shown that excessive prolonged exposure to stressors plays a critical role in the development and maintenance of many diseases (e.g., cardiovascular or metabolic diseases) and mental or neurodegenerative disorders such as depression, anxiety, or chronic fatigue syndrome (CFS) [1, 2].

Stress is a state of threatened homeostasis that is caused by internal or external adverse forces, so-called stressors (e.g., psychological, environmental, or physiological factors). Stressful events result in multiple neurochemical, neurotransmitter, and hormonal changes by primarily activating the sympathetic nervous system (SNS) and hypothalamic–pituitary–adrenal (HPA) axis [2, 3]. In reaction to stress, there is an increased release of hormones such as glucocorticoids, catecholamines, growth hormones, and prolactin, mobilizing energy sources and adapting the individual to his new circumstances. The most important targets of these hormones include the cognitive, reward, and fear system, as well as the gastrointestinal, cardiovascular, metabolic, and immune systems. Insufficient functionality of this stress system, dysregulation, or chronic activity, can harm the organism, affecting development, growth, and body composition, and lead to a variety of behavioral and somatic pathological conditions [3, 4].

In addition to these inflammatory and metabolic processes, mechanisms are activated in response to stress at the cellular level, which leads to the repair or elimination of stress-damaged macromolecules such as proteins, lipids but also DNA, and RNA, and these can trigger apoptosis [5]. Stress reactions are also epigenetically regulated and thus affect gene expression and post-transcriptional mechanisms. Besides, an altered stress-induced gene expression can lead to premature aging, inflammation, oxidative damage to various organs, or, in the long term, to burnout, depression, or CFS [6, 7]. Furthermore, there is growing evidence that non-coding RNAs, especially miRNAs, play an important role in physiological and psychological stress responses. These short RNAs with a length of 22–23 nucleotides control the expression of many protein-coding genes, which are responsible for cellular processes such as cell growth, proliferation, differentiation, and apoptosis. MiRNAs can affect multiple targets by binding to messenger RNAs (mRNAs) and inducing their cleavage, degradation, or translation repression [5, 7]. Animal studies have shown that changes in miRNA expression levels are functional responses to psychological stress and that these changes depend on the stressor (acute or chronic). Acute stress led to a significant increase in let-7a, whereas elevated miR-15a levels are associated with chronic psychological stress [7, 8]. MiRNAs can be identified in several body fluids and can provide information about various processes throughout the body, because miRNAs are mobile and can travel from one tissue to another, e.g., via extracellular vesicles [9]. This is one of the main reasons that miRNAs could make ideal candidates for biomarkers in a variety of diseases. For example, there is evidence that altered miRNA expression in serum, plasma, and cerebrospinal fluid is an early indication of pathological changes in the brain involved in mood disorders [10]. Compared to traditional biomarkers, e.g., proteins, miRNAs would have the advantage that the development of new assays takes less time and is less expensive than the generation of new antibodies for protein markers. They can be easily extracted from blood, urine, and other body fluids, and can be used as multimarker panels, improving the reliability of a diagnosis, while testing many proteins is more expensive and time-consuming [11].

Since stress is an important cofactor in the development and maintenance of many acute and chronic diseases, stress is of high epidemiological importance. It is of relevance to health economics and should be recognized at an early stage before health consequences occur. Since miRNAs are involved in the epigenetic regulation of stress reactions, they can be used as biomarkers in the diagnosis, prevention, and therapy evaluation of stress-associated diseases and mental health issues [7, 10]. The main aim of this study was to identify miRNAs in dried capillary blood spot (DBS) samples that are associated with stress-related diseases (or chronic stress) or the stress of a person and could be used as minimally invasive biomarkers. Capillary blood is a viable alternative to venous blood and studies have shown that the components (e.g., red blood cells) between capillary and venous blood are similar and comparable [12, 13]. Also, capillary blood is more stable than saliva because saliva is strongly influenced by lifestyle, such as smoking. Studies have shown that smokers have altered miRNA expression (e.g., miR-142) in saliva compared to non-smokers [14]. However, lifestyle interventions such as regular physical activity, certain diets, or foods (e.g., phytochemicals) have been shown to have a positive effect on mental and general health and can change miRNA expression [15, 16]. These may also have a positive effect on stress-associated miRNAs and could possibly be suggested as an additional therapy. Certain food could have protective effects on mental health via miRNAs. We therefore also examined the role of diet and other lifestyle factors, such as exercise, as it is known that mood or mental health is closely related to diet [17].

## Materials and methods

### Study design

For this observational study, we interviewed 173 individuals, 36.4% males, and 63.6% females, about their lifestyle and eating habits, with a special focus on their stress levels and stress-related diseases (SRDs) or symptoms, which are indicative of chronic stress, such as chronic fatigue syndrome (CFS), sleep disorders, mood swings, headaches/migraines (H/M), depression, and anxiety. From all study participants, capillary blood (DBS) and a standardized questionnaire were collected. The conduct of the study was approved by the Vienna Ethics Committee (MA 15—EK/22–091–VK_NZ) and all participants gave their written consent following the Declaration of Helsinki to the use of their data. The information provided by the study participants were treated confidentially throughout the process and the general data protection regulations were complied with. Table 1 gives an overview of all participants and their stress levels or stress-related diseases.

**Table 1:** Overview of the participants including their self-reported current stress levels and stress-related symptoms or diseases (SRDs). Participants could indicate several SRDs and a total of 83 reported having one or more SRDs. Age and BMI data were given as mean ± standard deviation (SD)

	Male	Female	Total
*n* (%)	63 (36.4%)	110 (63.6%)	173 (100%)
Age [years]	48.30 ± 13.08	50.47 ± 15.11	49.68 ± 14.41
Age range [years]	22–77	18–84	18–84
BMI [kg/m^2^]^*^	27.18 ± 4.74	24.53 ± 5.10	25.43 ± 5.12
BMI range [kg/m^2^]	20.45–46.75	17.26–43.51	17.26–46.75
Stress level distribution:			
Low % (*n*)	25.4% (16)	26.4% (29)	26.0% (45)
Moderate % (*n*)	15.9% (10)	7.3% (8)	10.4% (18)
High % (*n*)	42.9% (27)	44.5% (49)	43.9% (76)
Very high % (*n*)	15.9% (10)	21.8% (24)	19.7% (34)
Stress-related diseases (SRDs):			
Depression % (*n*)	9.5% (6)	9.1% (10)	9.2% (16)
Anxiety % (*n*)	23.8% (15)	26.4% (29)	25.4% (44)
Chronic fatigue syndrome (CFS) % (*n*)	30.2% (19)	26.4% (29)	27.7% (48)
Mood swings % (*n*)	3.2% (2)	3.6% (4)	3.5% (6)
Headaches or migraines % (*n*)	3.2% (2)	9.1% (10)	6.9% (12)
Sleep disorder % (*n*)	1.6% (1)	3.6% (4)	2.9% (5)
Total participants with at least one SRD % (*n*)	46.0% (29)	49.1% (54)	48.0% (83)

* *p* ≤ 0.05, indicating a difference between male and female participants

It is known that a certain level of stress, low and moderate, can have protective effects, while excessive or chronic stress is involved in the maintenance and development of numerous diseases (e.g., depression, burnout, or Alzheimer’s disease) [15]. We, therefore, divided the participants into two groups based on their self-reported stress levels to identify stress-related miRNA expression profiles that are primarily involved in excessive stress that the body can no longer cope with. All participants reporting high or very high-stress levels were assigned to the “stress group” (*n* = 110) and all participants reporting low or moderate stress levels and no SRDs were assigned to the “control group” (*n* = 47; Table 2). Participants who did not indicate their stress level or reported that they had low-stress levels but had an SRD were not assigned to either group (*n* = 16) but were included in the analysis of miRNAs and SRDs.

**Table 2:** Overview of the participants of the stress and control group. Age and BMI data were given as mean ± standard deviation (SD)

	Stress group	Control group
*n* (%)	110 (63.6%)	47 (27.2%)
Male *n* (%)	37 (33.6%)	20 (42.6%)
Female *n* (%)	73 (66.4%)	27 (57.4%)
Age [years]	47.81 ± 13.16	53.62 ± 16.55
Age range [years]	18–84	19–79
BMI [kg/m^2^]	24.52 ± 4.17	25.20 ± 4.24
BMI range [kg/m^2^]	17.26–37.13	18.82–39.26

### Sample collection and RNA-extraction

Capillary blood was collected on *Whatman® protein saver cards* (Sigma-Aldrich, Vienna, Austria) using *Safety Lancet Extra 18G* (Sarstedt, Nümbrecht, Germany), dried, and stored at room temperature. For the RNA extraction, the *MagMAX™ FFPE DNA/RNA Ultra Kit* (ThermoFisher Scientific, Waltham, MA, USA) was used. Four 4 mm points were punched out of each card, which were then incubated in protease solution at 55 °C overnight. The further extraction steps were carried out according to the manufacturer’s protocol. The RNA samples were stored at − 20 °C.

### cDNA synthesis and real-time quantitative PCR (qPCR)

*TaqMan™ Advanced microRNA cDNA Synthesis Kit* and *TaqMan™ Advanced microRNA assays* were used under the default settings on *QuantStudio™ 3* from ThermoFisher Scientific, Waltham, MA, USA. The cDNA samples were stored at − 20 °C. Based on literature research [7, 18–20], the following 13 miRNAs were analyzed: miR-10a-5p, miR-15a-5p, miR-16-5p, miR-19b-3p, miR-26b-5p, miR-29c-3p, miR-106b-5p, miR-126-3p, miR-142-3p, let-7a-5p, let-7g-5p, miR-21-5p, and miR-877-5p, as well as two endogenous controls (miR-24-3p and miR-93-5p). ∆Ct was used to evaluate the expression pattern and calculated as follows:

$$\Delta Ct{\mkern 1mu} = {\mkern 1mu} Ct^{{target}} {\mkern 1mu} - {\mkern 1mu} Ct^{{endogenous\,control}}$$

[21].

### Statistical analysis

The programs *IBM SPSS statistics 20* and *GraphPad Prism 6* were used for statistical analysis. All data are represented as mean ± standard deviation (SD). To test whether there is a difference between stress and no stress or between SRDs and no SRDs the independent t-test was used, ANCOVA to account for covariates for parametric and the Quade’s ANCOVA and Mann–Whitney-U test for nonparametric values. Multiple linear regression analyses were used to determine the miRNA predictive power of stress. Differences between women and men in self-reported variables (e.g., stress level or SRDs) were assessed using Chi-square test and independent t-test and miRNA Expression using independent t-test and ANOCA, including Quade’s ANCOVA and Mann–Whitney-U test. Correlations between nutritional or lifestyle habits and miRNA expressions were tested using Pearson’s or Spearman’s Rho correlation and Kendall’s Tau. A *p*-value ≤ 0.05 was assumed to be significant for all tests.

## Results

### Evaluation of stress-related miRNA expression profiles

**Fig. 1:**
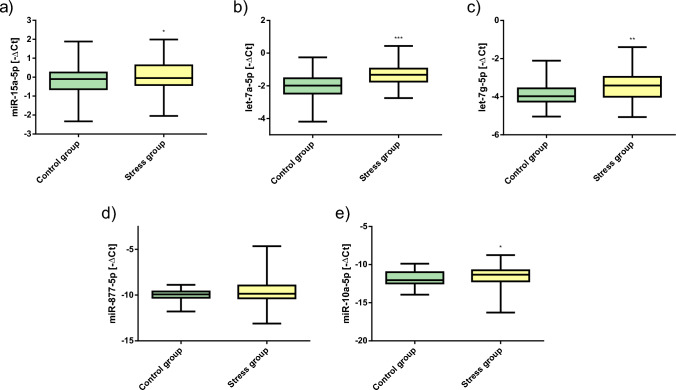
Stress-related miRNA expression profiles. **a-e** Boxplots of miRNA expression for **a** miR-15a-5p, **b** let-7a-5p, **c** let-7g-5p, **d** miR-877-5p, and **e** miR-10a-5p. The results are expressed as mean ± standard deviation (SD). ^*^p < 0.05, ^**^ *p* < 0.01, ^***^ *p* < 0.001 (ANCOVA)

There were no statistically significant sex differences in self-reported stress levels and SRDs (*p* > 0.05). Women and men differed only in BMI (*p* = 0.002; Table 1, and Table S1). Four of the analyzed miRNAs were expressed significantly different between the two groups (independent t-test, Table 3). Stress group participants had a significantly higher expression of miR-15a, let-7a, let-7g, and miR-877 than those in the control group (Fig. 1).

**Table 3:** Overview of the results of stress-related miRNA expression profiling. Expression data were presented as mean ± standard deviation (SD)

Marker	independent t-test *p*-value	ANCOVA *p*-value (covariate = age + BMI)	Hedges’ *g*	Stress group *n* = 110 mean ∆Ct ± SD	Control group *n* = 47 mean ∆Ct ± SD
miR-10a-5p	0.067	0.020^*^	0.406	11.417 ± 1.031	11.840 ± 1.081
miR-15a-5p	0.025^*^	0.040^*^	0.396	− 0.078 ± 0.754	0.205 ± 0.609
let-7a-5p	0.000^*^	0.000^*^	1.073	1.348 ± 0.599	2.038 ± 0.734
let-7g-5p	0.000^*^	0.009^*^	0.626	3.459 ± 0.753	3.891 ± 0.518
miR-877-5p	0.018^*^	0.117	0.343	9.619 ± 1.278	10.008 ± 0.704
miR-16-5p	0.091	0.174	0.267	− 3.971 ± 0.625	− 3.783 ± 0.648
miR-19b-3p	0.390	0.120	0.189	2.150 ± 1.069	2.355 ± 1.143
miR-26b-5p	0.428	0.315	0.173	1.625 ± 0.762	1.759 ± 0.814
miR-29c-3p	0.308	0.172	0.223	5.083 ± 0.806	5.261 ± 0.763
miR-106b-5p	0.302	0.429	0.182	3.294 ± 0.676	3.420 ± 0.734
miR-126-3p	0.571	0.406	0.124	1.466 ± 0.736	1.554 ± 0.601
miR-142-3p	0.391	0.442	0.114	4.248 ± 0.822	4.340 ± 0.742
miR-21-5p	0.519	0.723	0.032	3.743 ± 0.662	3.720 ± 0.811

*Significant at *p* ≤ 0.05

However, since the two groups differ in terms of age (*p* = 0.021) but not in BMI (*p* = 0.367), see Table 2. We additionally analyzed the measured miRNAs using ANCOVA and age and BMI as covariates. The ANCOVA showed significant differences between the groups in the expression of miR-10a, miR-15a, let-7a, and let-7g (Table 3 and Fig. 1). Examining the results of multiple linear regression analyses (Table S2), showed that stress is a significant predictor of the expression of miR-10a (*p* = 0.020), miR-15a (*p* = 0.040), let-7a (*p* = 0.000), and let-7g (*p* = 0.006). According to the results of the analysis, the regression equations are as follows:

$$miR10a \left[\Delta Ct\right]=12.844-\left(0.594\times group\right)-\left(0.019\times age\right)+(0.004\times BMI)$$

$$miR15a \left[\Delta Ct\right]=-0.267-\left(0.279\times group\right)-\left(0.002\times age\right)+(0.022\times BMI)$$

$$let7a \left[\Delta Ct\right]=1.338-\left(0.653\times group\right)+\left(0.008\times age\right)+(0.012\times BMI)$$

$$let7g \left[\Delta Ct\right]=2.587-\left(0.362\times group\right)+\left(0.008\times age\right)+(0.020\times BMI)$$

The model showed that stressed individuals had 0.594 higher expression of miR-10a, 0.279 higher expression of miR-15a, 0.653 higher expression of let-7a, and 0.362 higher expression of let-7g.

### MiRNAs associated with stress-related diseases (SRDs)

**Fig. 2:**
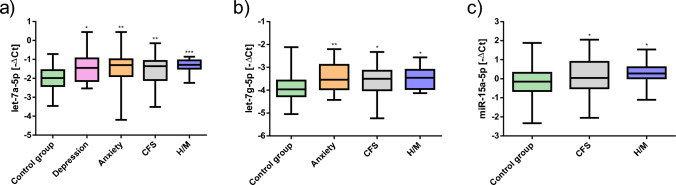
Specific miRNA expression levels for stress-related diseases (SRDs). (**a-c**) Boxplots of miRNA expression for **a** let-7a-5p, **b** let-7g-5p, and **c** miR-15a-5p. The results are expressed as mean ± standard deviation (SD). ^*^p < 0.05, ^**^ *p* < 0.01, ^***^ *p* < 0.001 (independent t-test or ANCOVA)

83 participants reported having one or more SRDs (Table 1). Participants who reported suffering from depression had significantly higher let-7a expression levels compared to the control group (Fig. 2a). Participants reporting anxiety had significantly higher expression levels of let-7a (Fig. 2a) and let-7g (Fig. 2b). Participants who stated suffering from chronic fatigue syndrome (CFS) and participants with headaches/migraines (H/M) also had higher expression levels of let-7a (Fig. 2a), let-7g (Fig. 2b), and miR-15a (Fig. 2c). The mean miRNA expressions of the respective SRDs can be found in the supplementary material Table S3.

### Correlations between miRNAs and nutritional or lifestyle habits

We discovered a negative correlation between the number of SRDs mentioned by a participant and the BMI (one *n* = 50, mean BMI = 26.82 kg/m^2^; two *n* = 20, mean BMI = 23.95 kg/m^2^; ≥ three *n* = 13, mean BMI = 22.38 kg/m^2^; *p* = 0.024). The BMI reduced significantly the more SRDs a participant reported. Additionally, an association between meat consumption and let-7a was observed. The more meat consumed per week, the higher the expression of let-7a (rarely/never *n* = 43, mean ∆Ct = 1.822; 1–6 portions/week *n* = 114, mean ∆Ct = 1.524; ≥ everyday *n* = 7, mean ∆Ct = 1.484; *p* = 0.031). Furthermore, participants who reported that they regularly consumed coffee (≥ 1 cup/day, *n* = 137, mean ∆Ct = 0.027) had a lower expression of miR-15a than those who stated that they never consume coffee (*n* = 33, mean ∆Ct = − 0.257; *p* = 0.028).

## Discussion

The identification of new biomarkers to assess acute or chronic psychological stress could not only provide helpful information for the prevention and treatment of stress-related disorders but also support overall health or well-being and healthy aging. Therefore, this study aimed to determine miRNAs that are suitable for assessing personal stress levels and detecting stress-related consequences before physical impairments occur. Four possible candidates could be figured out in this study: miR-10a, miR-15a, let-7a, and let-7g. These miRNAs showed higher expression levels in stressed individuals than in non-stressed, and three (let-7a, let-7g, and miR-15a) of them correlated with SRDs, indicating long-term consequences of chronic stress.

Many miRNAs are discussed in the context of psychological stress, anxiety, depression, and other SRDs [7]. It is hypothesized that changes in miRNA expression induced by environmental stressors result in altered neuronal morphology and problems with neuronal circuitry leading to relevant health problems. An increase of let-7a expression in the brain and serum of mice could be observed after exposure to acute obsessive–compulsive stress, which only returned to its initial level five days later [7, 22]. Upregulation of this miRNA could also be measured in rats after an acute social defeat [23]. These results and our findings in human capillary blood support the hypothesis that let-7a would be suitable as a biomarker to detect acute stress. Furthermore, it was discovered that several members of the let-7 family, including let-7a, are potent activators of toll-like receptors (TLRs) signaling in microglia and neurons, and that TLR activation induces dose- and time-dependent neuronal cell death, which is associated with neurodegeneration and pathogenesis of neurodegenerative diseases [24].

MiR-10a is also considered a possible biomarker for acute psychological stress [7]. In line with our observations, acute stress induction resulted in the upregulation of miR-10a in whole blood and appears to correlate with stress-induced alcohol consumption [25]. Additionally, overexpression of miR-10a downregulates the expression of Brain-derived neurotrophic factor (*BDNF*) in neurons and low expression levels of *BDNF* are hypothesized to play an important role in the pathogenesis of major depression disorder (MDD) [26]. Another link between miR-10a and stress is SIRT1. Overexpression of miR-10a leads to downregulation of *SIRT1* expression, which is also associated with MDD [26].

Studies suggest that miR-15a plays a role in chronic stress management mechanisms. Animal models have shown elevated levels of miR-15a in the blood of healthy subjects who suffered childhood trauma (CT) compared to subjects without CT [27]. Moreover, let-7g could be linked to CT [19]. MiR-15a appears to regulate serotonin transporter (*SERT*) expression through binding to the gene *SLC6A4*. Altered *SERT* expression is associated with a variety of diseases such as anxiety/obsessive–compulsive disorders, depression, or autism [28]. In this study, we were able to identify connections between miR-15a, let-7g, and SRDs (e.g., CFS or anxiety). However, this result by itself is not strong enough to identify SRDs using two miRNAs and requires more precise pathway analysis with the measurement of stress-related hormones. Nevertheless, it provides the first evidence that miR-15a and let-7g are involved in chronic stress mechanisms.

Lifestyle and nutrition can affect brain (dys-)function, presumably through influencing inflammation [29]. Our data indicated possible effects of lifestyle and dietary habits on miRNAs, which are also involved in stress regulation. Participants who regularly consumed meat showed higher let-7a expression than those who rarely or never consumed meat. Also, dietary amino acids, such as those in meat, form the major monoamines (e.g., norepinephrine, dopamine, and serotonin), affecting mental health [17]. It can be assumed that let-7a is also involved in this regulation. Furthermore, we could observe a correlation between miR-15a expression and coffee consumption. Coffee contains many bioactive compounds or phytochemicals such as polyphenols, polysaccharides, vitamins, and minerals that have health-promoting (e.g., antioxidant, and anti-inflammatory) properties [29, 30]. While the literature describes coffee as inappropriately activating the sympathetic nervous system, caffeine is associated with psychological distress. Coffee has further health benefits, such as improved heart and brain functions [17]. Polyphenols are known to have certain regulators, e.g., miRNAs, that affect gene expression [31], which could explain our observed associations between miRNAs and nutrition.

The findings of this study should be considered in light of some limitations. Since this is an observational study based on participants’ self-reports on stress levels and lifestyle and dietary habits, we could only report correlational relationships and speculate on the pathways behind potential biomarkers. However, our results are based on a relatively large number of study participants and thus offer valuable insights into the possibility of using a minimally invasive method to monitor a person’s health.

## Conclusion

Taken together, our results indicate that miR-10a and let-7a are pointing towards acute stress, whereas miR-15a, and let-7g towards chronic stress and may be suitable as new biomarkers for assessing mental health. Furthermore, our study provides some evidence that miRNAs from human capillary blood could be used as a cost-effective monitoring tool for the prevention of stress-related diseases in the future. However, some nutritional factors (e.g., meat or coffee) also play an important role in mental health and should be considered to maintain overall health.

## Supplementary Information

Below is the link to the electronic supplementary material.

Supplementary file1 (DOC 86 KB)

## Abbreviations

BDNF: Brain-derived neurotrophic factor
CFS: Chronic fatigue syndrome
CT: Childhood trauma
DBS: Dried capillary blood spot
HPA: Hypothalamic-pituitary-adrenal
MDD: Major depression disorder
qPCR: Real-time quantitative polymerase chain reaction
SERT: Serotonin transporter
SNS: Sympathetic nervous system
SRDs: Stress-related diseases
TLRs: Toll-like receptors

## References

[1] Kemeny ME (2003). The psychobiology of stress. Curr Dir Psychol Sci.

[2] Liu YZ, Wang YX, Jiang CL (2017). Inflammation: the common pathway of stress-related diseases. Front Hum Neurosci.

[3] Chrousos GP (2009). Stress and disorders of the stress system. Nat Rev Endocrinol Nature Publishing Group.

[4] Ranabir S, Reetu K (2011). Stress and hormones. Indian J Endocrinol Metab.

[5] Olejniczak M, Kotowska-Zimmer A, Krzyzosiak W (2018). Stress-induced changes in MiRNA biogenesis and functioning. Cell Mol Life Sci Springer Int Publ.

[6] Park C, Rosenblat JD, Brietzke E, Pan Z, Lee Y, Cao B, Zuckerman H, Kalantarova A, McIntyre RS (2019). Stress, epigenetics and depression: a systematic review. Neurosci Biobehav Rev Elsevier Ltd.

[7] Wiegand C, Savelsbergh A, Heusser P (2017). MicroRNAs in psychological stress reactions and their use as stress-associated biomarkers, especially in human saliva. Biomed Hub.

[8] Volk N, Pape JC, Engel M, Zannas AS, Cattane N, Cattaneo A, Binder EB, Chen A (2016). Amygdalar MicroRNA-15a Is essential for coping with chronic stress. Cell Rep ElsevierCompany.

[9] Mori MA, Ludwig RG, Garcia-Martin R, Brandão BB, Kahn CR (2019). Extracellular MiRNAs: from biomarkers to mediators of physiology and disease. Cell Metab.

[10] Roy B, Ochi S (2023). Potential of circulating MiRNAs as molecular markers in mood disorders and associated suicidal behavior. Int J Mol Sci.

[11] Condrat CE, Thompson DC, Barbu MG, Bugnar OL, Boboc A, Cretoiu D, Suciu N, Cretoiu SM, Voinea SC (2020). MiRNAs as biomarkers in disease: latest findings regarding their role in diagnosis and prognosis. Cells.

[12] Simmonds MJ, Baskurt OK, Meiselman HJ, Marshall-Gradisnik SM (2011). A comparison of capillary and venous blood sampling methods for the use in haemorheology studies. Clin Hemorheol Microcirc.

[13] Krammer UDB, Tschida S, Berner J, Lilja S, Switzeny OJ, Hippe B, Rust P, Halsberger AG (2022). MiRNA-based “fitness score” to assess the individual response to diet, metabolism and exercise. J Int Soc Sports Nutr.

[14] Öngöz Dede F, Gökmenoğlu C, Türkmen E, Bozkurt Doğan Ş, Sertaç Ayhan B, Yildirim K (2023). Six MiRNA expressions in the saliva of smokers and non-smokers with periodontal disease. J Periodontal Res.

[15] Rippe JM (2018). Lifestyle medicine: the health promoting power of daily habits and practices. Am J Lifestyle Med.

[16] Flowers E, Won GY, Fukuoka Y (2015). Micrornas associated with exercise and diet: a systematic review. Physiol Genomics.

[17] Begdache L, Chaar M, Sabounchi N, Kianmehr H (2019). Assessment of dietary factors, dietary practices and exercise on mental distress in young adults versus matured adults: a cross-sectional study. Nutr Neurosci Taylor Francis.

[18] Katsuura S, Kuwano Y, Yamagishi N, Kurokawa K, Kajita K, Akaike Y, Nishida K, Masuda K, Tanahashi T, Rokutan K (2012). MicroRNAs MiR-144/144* and MiR-16 in peripheral blood are potential biomarkers for naturalistic stress in healthy Japanese medical students. Neurosci Lett Elsevier Ireland Ltd.

[19] Van der Auwera S, Ameling S, Wittfeld K, d’Harcourt Rowold E, Nauck M, Völzke H, Suhre K, Najafi-Shoushtari H, Methew J, Ramachandran V, Bülow R, Völker U, Grabe HJ (2019). Association of childhood traumatization and neuropsychiatric outcomes with altered plasma Micro RNA-levels. Neuropsychopharmacology.

[20] Hippe B, Krammer U, Mödder F, Mayer A, Speich S, Gruber U, Jacob U, Haslberger A (2022). Epigenetic Active phytoceuticals activate immune relevant MiRNAs important in virus response systems. Funct Foods Heal Dis.

[21] Krammer UDB, Sommer A, Tschida S, Mayer A, Lilja SV, Switzeny OJ, Hippe B, Rust P, Haslberger AG (2022). PGC- 1α methylation, MiR-23a, and MiR-30e expression as biomarkers for exercise- and diet-induced mitochondrial biogenesis in capillary blood from healthy individuals: a single-arm intervention. Sports.

[22] Sung M, Sung SE, Kang KK, Choi JH, Lee S, Kim K, Lim JH, Lee GW, Rim HD, Kim BS, Won S, Kim K, Jang S, Seo MS, Woo J (2021). Serum-derived neuronal exosomal mirnas as biomarkers of acute severe stress. Int J Mol Sci.

[23] Chen RJ, Kelly G, Sengupta A, Heydendael W, Nicholas B, Beltrami S, Luz S, Peixoto L, Abel T, Bhatnagar S (2015). MicroRNAs as biomarkers of resilience or vulnerability to stress. Neuroscience IBRO.

[24] Hollins SL, Cairns MJ (2016). MicroRNA: small RNA mediators of the brains genomic response to environmental stress. Prog Neurobiol Elsevier Ltd.

[25] Beech RD, Leffert JJ, Lin A, Hong KA, Hansen J, Umlauf S, Mane S, Zhao H, Sinha R (2014). Stress-related alcohol consumption in heavy drinkers correlates with expression of MiR-10a, MiR-21, and components of the TAR-RNA-binding protein-associated complex. Alcohol Clin Exp Res.

[26] Wan Y, Liu Y, Wang X, Wu J, Liu K, Zhou J, Liu L, Zhang C (2015). Identification of differential MicroRNAs in cerebrospinal fluid and serum of patients with major depressive disorder. PLoS ONE.

[27] Maffioletti E, Bocchio-Chiavetto L, Perusi G, Carvalho Silva R, Sacco C, Bazzanella R, Zampieri E, Bortolomasi M, Gennarelli M, Minelli A (2021). Inflammation-related MicroRNAs are involved in stressful life events exposure and in trauma-focused psychotherapy in treatment-resistant depressed patients. Eur J Psychotraumatol Taylor Francis.

[28] Moya PR, Wendland JR, Salemme J, Fried RL, Murphy DL (2013). MiR-15a and MiR-16egulate serotonin transporter expression in human placental and rat brain raphe cells. Int J Neuropsychopharmacol.

[29] de Melo Pereira GV, de Carvalho Neto DP, Magalhães AI, do Prado FG, Pagnoncelli MGB, Karp SG, Soccol CR (2020). Chemical composition and health properties of coffee and coffee by-products. Adv Food Nutr Res.

[30] Samsonowicz M, Regulska E, Karpowicz D, Leśniewska B (2019). Antioxidant properties of coffee substitutes rich in polyphenols and minerals. Food Chem.

[31] Milenkovic D, Jude B, Morand C (2013). MiRNA as molecular target of polyphenols underlying their biological effects. Free Radic Biol Med Elsevier.

