# Supplementary material for: MiR-10a, miR-15a, let-7a, and let-7g expression as stress-relevant biomarkers to assess acute or chronic psychological stress and mental health in human capillary blood
Source: Mol Biol Rep. 2023 May 17;50(7):5647–54. doi: 10.1007/s11033-023-08467-5 (PMC10289991; doi:10.1007/s11033-023-08467-5)
Supplement: Supplementary file 1 — Supplementary file1 (DOC 86 KB) [file 11033_2023_8467_MOESM1_ESM.doc]

**Supplementary material**

**Table S1. Sex-specific characteristics in the respective groups. Age, BMI, and expression data were presented as mean ± standard deviation (SD).**

|  | **Stress group** | | | | **Control group** | | | |
| --- | --- | --- | --- | --- | --- | --- | --- | --- |
|  | **Male** (*n* = 37)  mean ∆Ct ± SD | **Female** (*n* = 73)  mean ∆Ct ± SD | **independent t-test**  *p*-value | **ANCOVA**  *p*-value | **Male** (*n* = 20)  mean ∆Ct ± SD | **Female** (*n* = 27)  mean ∆Ct ± SD | **independent t-test**  *p*-value | **ANCOVA**  *p*-value |
| miR-10a-5p | 11.476 ± 1.147 | 11.468 ± 1.157 | 0.973 | 0.414 | 11.937 ± 1.055 | 11.773 ± 1.127 | 0.706 | 0.473 |
| miR-15a-5p | -0.086 ± 0.752 | -0.075 ± 0.760 | 0.943 | 0.471 | 0.093 ± 0.473 | 0.210 ± 0.797 | 0.562 | 0.570 |
| let-7a-5p | 1.267 ± 0.554 | 1.389 ± 0.620 | 0.327 | 0.429 | 2.117 ± 0.580 | 1.976 ± 0.840 | 0.525 | 0.772 |
| let-7g-5p | 3.460 ± 0.616 | 3.459 ± 0.818 | 0.994 | 0.978 | 4,013 ± 0.490 | 3.797 ± 0.529 | 0.164 | 0.264 |
| miR-877-5p | 9.675 ± 1.631 | 9.590 ± 1.065 | 0.781 | 0.266 | 10.277 ± 0.761 | 9.800 ± 0.591 | 0.021^*^ | 0.016^*^ |
| miR-16-5p | -3.977 ± 0.636 | -3.968 ± 0.623 | 0.943 | 0.192 | -3.843 ± 0.566 | -3.739 ± 0.711 | 0.595 | 0.561 |
| miR-19b-3p | 2.195 ± 1.139 | 2.126 ± 1.040 | 0.770 | 0.636 | 2.274 ± 1.146 | 2.411 ± 1.176 | 0.766 | 0.871 |
| miR-26b-5p | 1.499 ± 0.668 | 1.692 ± 0.806 | 0.251 | 0.071 | 1.675 ± 0.649 | 1.817 ± 0.926 | 0.665 | 0.832 |
| miR-29c-3p | 5.084 ± 0.773 | 5.082 ± 0.829 | 0.993 | 0.378 | 5.213 ± 0.674 | 5.295 ± 0.839 | 0.792 | 0.903 |
| miR-106b-5p | 3.186 ± 0.595 | 3.348 ± 0.711 | 0.234 | 0.061 | 3.403 ± 0.642 | 3.431 ± 0.804 | 0.901 | 0.485 |
| miR-126-3p | 1.461 ± 0.642 | 1.468 ± 0.786 | 0.965 | 0.408 | 1,654 ± 0.513 | 1.484 ± 0.662 | 0.481 | 0.470 |
| miR-142-3p | 4.140 ± 0.824 | 4.306 ± 0.822 | 0.359 | 0.167 | 4.434 ± 0.662 | 4.376 ± 0.813 | 0.845 | 0.791 |
| miR-21-5p | 3.735 ± 0.706 | 3.747 ± 0.644 | 0.933 | 0.419 | 3.841 ± 0.471 | 3.798 ± 0.540 | 0.790 | 0.251 |
| Age [years] | 45.08 ± 10.07 | 49.19 ± 14.34 | 0.122 |  | 53.90 ± 15.44 | 53.41 ± 17.62 | 0.921 |  |
| BMI [kg/m²] | 25.68 ± 2.87 | 24.00 ± 4.56 | 0.027* |  | 26.97 ± 3.50 | 23.98 ± 4.34 | 0.019* |  |

*Significant at p ≤ 0.05. Covariates are age and BMI.

**Table S2. Results of multiple linear regression analyses.**

| **Predictive variable** | **Predicted variable (miR-10a [∆Ct])** | | | | | | | | |
| --- | --- | --- | --- | --- | --- | --- | --- | --- | --- |
|  | *B* | Standard error | | | *β* | | *t* | | *p-value* |
| Constant | 12.844 | 0.741 | | |  | | 17.324 | | 0.000 |
| BMI [kg/m2] | 0.004 | 0.024 | | | 0.016 | | 0.174 | | 0.863 |
| Age [years] | -0.019 | 0.008 | | | -0.234 | | -2.415 | | 0.017^*^ |
| Group | -0.594 | 0.252 | | | -0.227 | | -2.355 | | 0.020^*^ |
| R = 0.208, R^2^ = 0.079, *p* = 0.032, *f* = 0.293 | | | | | | | | | |
|  | **Predicted variable (miR-15a [∆Ct])** | | | | | | | | |
| Constant | -0.267 | 0.315 | | |  | | 2.440 | | 0.521 |
| BMI [kg/m2] | 0.022 | 0.015 | | | 0.126 | | 1.502 | | 0.135 |
| Age [years] | -0.002 | 0.004 | | | -0,037 | | -0.434 | | 0.665 |
| Group | -0.279 | 0.135 | | | -0,174 | | -2.072 | | 0.040^*^ |
| R = 0.216, R^2^ = 0.047, *p* = 0.081, *f* = 0.222 | | | | | | | | | |
|  | **Predicted variable (let-7a [∆Ct])** | | | | | | | | |
| Constant | 1.338 | | 0.381 |  | | 3.514 | | 0.001 | |
| BMI [kg/m2] | 0.012 | | 0.013 | 0.067 | | 0.873 | | 0.381 | |
| Age [years] | 0.008 | | 0.004 | 0.149 | | 1.915 | | 0.058 | |
| Group | -0.653 | | 0.121 | -0.415 | | -5.386 | | 0.000^*^ | |
| R = 0.483, R^2^ = 0.233, *p* = 0.000, *f* = 0.551 | | | | | | | | | |
|  | **Predicted variable (let-7g [∆Ct])** | | | | | | | | |
| Constant | 2.948 | | 0.408 |  | | 7.235 | | 0.000 | |
| BMI [kg/m2] | 0.020 | | 0.014 | 0.116 | | 1.410 | | 0.161 | |
| Age [years] | 0.008 | | 0.004 | 0.150 | | 1.786 | | 0.076 | |
| Group | -0.362 | | 0.130 | -2.31 | | -2.789 | | 0.006^*^ | |

R = 0.337, R^2^ = 0.114, p = 0.001, f = 0,359; ^*^Significant at p ≤ 0.05.

**Table S3.** MiRNA expression patterns of the different stress-related diseases (SRDs). Expression data were presented as mean ± standard deviation (SD).

|  | **Control group**  mean ∆Ct ± SD | **Depression**  mean ∆Ct ± SD | **Anxiety**  mean ∆Ct ± SD | **CFS**  mean ∆Ct ± SD | **H/M**  mean ∆Ct ± SD |
| --- | --- | --- | --- | --- | --- |
| *n* | 47 | 16 | 44 | 48 | 12 |
| let-7a-5p | 2.038 ± 0.734 | 1.531 ± 0.786 | 1.405 ± 0.786 | 1.545 ± 0.730 | 1.358 ± 0.406 |
| let-7g-5p | 3.891 ± 0.518 | - | 3.443 ± 0.621 | 3.542 ± 0.702 | 3.457 ± 0.493 |
| miR-15a-5p | 0.205 ± 0.609 | - | - | -0.184 ± 0.863 | -0.310 ± 0.634 |

- indicating no significant difference between the groups; CFS = chronic fatigue syndrome; H/M = headaches/migraines.
